# Peculiarities of γ-Al_2_O_3_ Crystallization on the Surface of h-BN Particles

**DOI:** 10.3390/ma15228054

**Published:** 2022-11-15

**Authors:** Sergey N. Grigoriev, Elena A. Trusova, Asya M. Afzal, Thet Naing Soe, Alexandra Yu. Kurmysheva, Ekaterina Kuznetsova, Anton Smirnov, Nestor Washington Solís Pinargote

**Affiliations:** 1 Laboratory of Electric Current Assisted Sintering Technologies, Moscow State University of Technology “STANKIN”, Vadkovsky per. 1, 127055 Moscow, Russia; 2 Baikov Institute of Metallurgy and Materias Science, RAS, Russian Academy of Sciences, Leninsky pr. 49, 119334 Moscow, Russia

**Keywords:** h-BN, nano-alumina, sol–gel, γ-Al_2_O_3_@h-BN, nanostructures, crystallization on surface, chemical homogeneity of composites

## Abstract

The main goal of the present work was to synthesize a composite consisting of h-BN particles coated with a γ-Al_2_O_3_ nanolayer. A method was proposed for applying nanocrystalline γ-Al_2_O_3_ to h-BN particles using a sol–gel technique, which ensures the chemical homogeneity of the composite at the nano level. It has been determined that during crystallization on the h-BN surface, the proportion of spinel in alumina decreases from 40 wt.% in pure γ-Al_2_O_3_ to 30 wt.% as a result of the involvement of the B^3+^ ions from the surface nitride monolayers into the transition complex. For comparison, nano-alumina was synthesized from the same sol under the same conditions as the composite. The characterization of the obtained nanostructured powders was carried out using TEM and XRD. A mechanism is proposed for the formation of a nanostructured γ-Al_2_O_3_@h-BN composite during the interaction of Al-containing sol and h-BN suspension in aqueous organic media. The resulting composite is a promising model of powdered raw materials for the development of fine-grained ceramic materials for a wide range of applications.

## 1. Introduction

Hexagonal boron nitride (h-BN) has a layered crystalline structure similar to that of graphite, which gives it high lubricity and machinability, resulting in it also being known as “white graphite” [1]. In addition to these characteristics, h-BN has other equally important properties, such as excellent chemical resistance, thermal stability, and good thermal conductivity with no electrical conductivity [2,3,4], that make it a promising candidate for use in lubrication applications. In recent years, the joint combination of these properties has attracted the attention of many researchers to use h-BN as a solid lubricant, which can successfully perform the self-lubricating functions of ceramic tools [5,6,7].

Ceramic cutting tools, due to their unique properties (high hardness [8] and excellent resistance to high temperatures [9,10], corrosion resistance [11], and wear resistance [12]), are used in the machining of hard materials and superalloys, as they can withstand extreme machining conditions [13,14]. However, ceramic tools have high coefficients of friction and are sensitive to defects generated during dry cutting [15,16,17]. Therefore, the addition of h-BN to the ceramic matrix can significantly increase its lubricity. During the cutting process, h-BN particles can release, smear, and develop a thin lubricating film, which effectively reduces the coefficient of friction and wear rate of the tool–workpiece interface [18,19]. On the other hand, the softness of the h-BN particle decreases the overall mechanical properties of ceramic composites [20]. An effective solution to maintain the lubricating properties and ensure the preservation of the mechanical properties of the ceramic compound is the formation of a protective layer on the surface of the h-BN particles [21,22,23]. For instance, Wu et al. [1] coated h-BN with a layer of Ni and showed that its addition to ceramic tools can avoid the negative effects on mechanical properties caused by the direct addition of h-BN. In another work, Chen et al. [24] compared to the addition of h-BN and h-BN@SiC to the Al_2_O_3_@TiC matrix and demonstrated that the inclusion of h-BN@SiC noticeably improved the mechanical properties and machinability of ceramic cutting tools.

Furthermore, it is known that the addition of h-BN leads to alumina toughening and prevents crack development, mainly by crack tip blunting [25,26,27,28]. Taking this fact into account and knowing that most ceramic cutting tools are made of alumina, it can be deduced that Al_2_O_3_ is an excellent material for h-BN coating. In addition, the coating of h-BN with Al_2_O_3_ can improve its interaction and dispersibility in the matrix of ceramic cutting tools [29]. This approach to improving the final product is common in many scientific fields [30,31,32].

We decided to combine the positive properties of h-BN and alumina in order to create a promising raw powder product for the sintering of fine-grained ceramics with improved properties. The potential market for such ceramics is almost comprehensive: from materials for miniaturized electronic devices operating under extreme conditions (high temperatures, vibration, mechanical stress) to blanks for cutting tools with improved performance characteristics (strength, wear resistance, durability). The creation of scientific foundations for the production technology of such materials will improve the accuracy of measuring instruments and cutting tools, optimize the costs of the reconstruction of existing industries, and increase the efficiency of production in general [33].

In this work, a mechanism for the formation of crystalline alumina on the surface of crystals is proposed, which explains a simple, economic, and fast method of obtaining h-BN@ γ-Al_2_O_3_ particles. In this method, nanocrystalline alumina can be deposited on h-BN nanocrystals using the sol–gel method. Furthermore, the influence of the h-BN crystal lattice on the morphology of Al_2_O_3_ and the proportion and syngony of spinel in its composition were also studied. The synthesized powders were characterized using X-ray diffraction (XRD) and transmission electron microscopy (TEM).

## 2. Materials and Methods

### 2.1. Preparation of Nanostructured γ-Al_2_O_3_@h-BN Powder

A nanolayer of γ-Al_2_O_3_ was deposited using the sol–gel method [34] on commercially available h-BN powder with an average particle size of 70 nm. The two-step γ-Al_2_O_3_ deposition process included obtaining a suspension of h-BN powder in a water–alcohol mixture with deionized water and isopropanol at a volume ratio of 2:1. The details of the purity and manufacturers of the reagents used are provided in Table 1. For the synthesis of the Al-containing sol, an aqueous solution of aluminum nitrate nonahydrate (Al(NO_3_)_3_·9H_2_O) at a concentration of 0.4 M was prepared. Then, in order to form and stabilize the sol, the monoethanolamine (MEA) was added to the Al(NO_3_)_3_ solution at an MEA/Al molar ratio of 3:1.

Once the h-BN suspension and the Al-containing sol were prepared, they were mixed through stirring (at 250 rpm) on a magnetic stirrer for 20–25 min and heated to 60–65 °C. Then, the mixture was evaporated with constant stirring at a temperature of 95–98 °C until a viscous mass was obtained. After that, the resulting mass was transferred into a porcelain cup and placed in a furnace, where heat treatment was carried out at 900 °C for 1 h. As a result, a fine white powder was synthesized with an h-BN content of 1.20 (±0.06) wt.%.

### 2.2. Characterization of Synthesized Powders

An X-ray diffractometer (SHIMADZU XRD-6000, Shimadzu, Japan) with monochromatic copper radiation (λKα = 1.54178 Å) and Zeeman–Bolin focusing was used to analyze the phase composition of the as-prepared particles. The determination of the phase composition was carried out using JCPDS cards. The studies were performed at room temperature and under normal atmospheric pressure. The average crystallite size was calculated using the Rietveld method, which uses an iterative procedure to minimize the experimental diffraction pattern deviations from the calculated data. Transmission electron microscopy (TEM) studies of obtained pure γ-Al_2_O_3_ were carried out with the use of an LEO-912 AB OMEGA (Leo Elektronenmikroskopie, Oberkochen, Germany) electron microscope operating at 100 kV.

## 3. Results and Discussion

**Figure 1:**
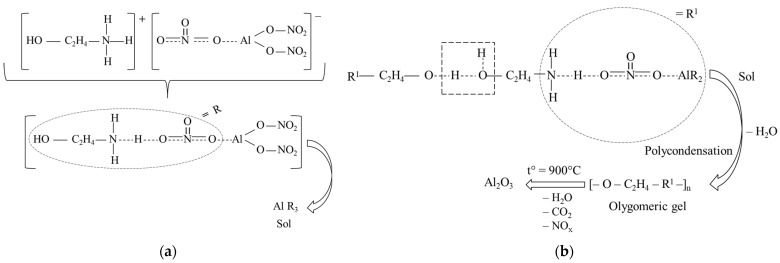
Schemes of (**a**) the formation of the Al-containing sol; and (**b**) the heat treatment of Al-containing sol within a wide temperature range, up to 900 °C, which includes the polycondensation of the complex sol and subsequent thermal destruction of the formed oligomeric gel, leading to the formation of nanocrystalline γ-Al_2_O_3_.

**Figure 2:**
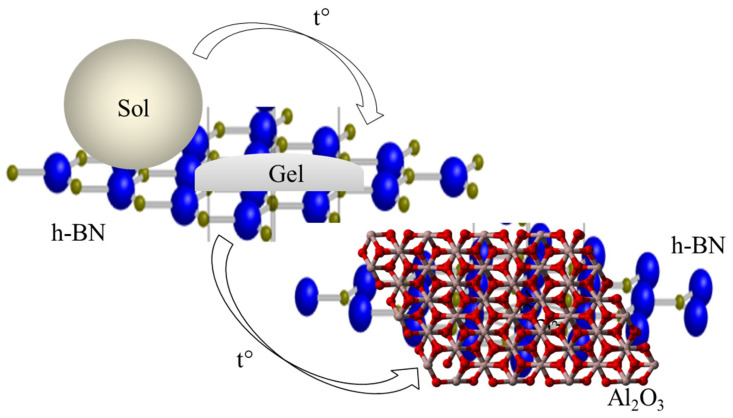
Scheme of the formation of the γ-Al_2_O_3_ crystal lattice on the surface of h-BN particles.

After MEA was added to the 0.4 M aqueous solution of aluminum nitrate, an Al-containing sol was formed as a result of the interaction of hydrolyzed MEA molecules and Al(NO_3_)_3_, as shown in Figure 1a. As is known, the hydrolysis of aluminum nitrate occurs in steps, and Figure 1a shows its first stage [34]. The quaternary ammonium ion and the triply charged nitrate anion were the centers of interaction between the MEA and aluminum nitrate. At an MEA/Al(NO_3_)_3_ molar ratio of 3:1, as a result of complexation, a sol with the overall formula of AlR_3_ was formed, and further, it was adsorbed on the surface of the crystalline h-BN particles. During the heat treatment (900 °C) of the resulting mixed colloid, the sol → gel transition (Figure 1b) occurs on the surface of h-BN crystalline particles. In this case, as a result of the polycondensation reaction, which was implemented at the first stage of heat treatment, an oligomeric gel was formed. The latter decomposed upon calcination in the air at 900 °C; this led to the crystallization of γ-Al_2_O_3_ on the centers of the surface of h-BN particles, which were formed earlier during the adsorption of the Al-containing sol on them (Figure 2).

The organic component of the gel was removed as water vapor and carbon and nitrogen oxides during the thermal degradation of the oligomeric gel (Figure 1b and Figure 2). The use of a high mass ratio of Al-containing sol and h-BN made it possible to create γ-Al_2_O_3_ shells on the particles of the latter.

**Figure 3:**
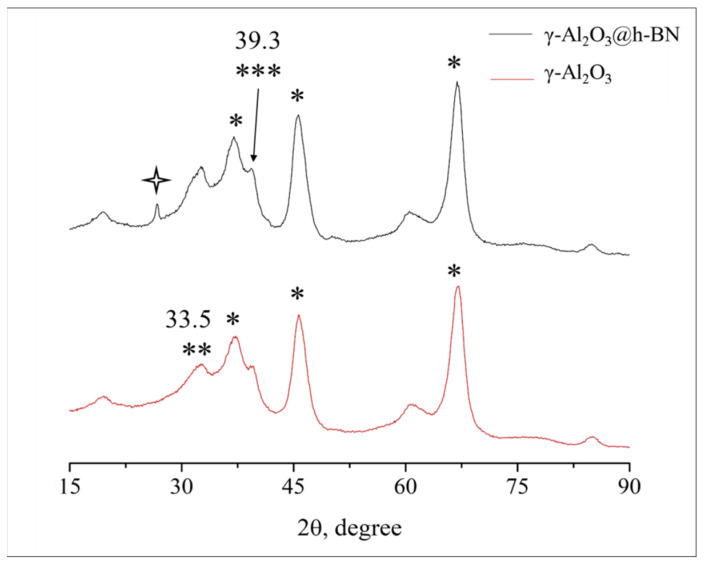
XRD patterns for the γ-Al_2_O_3_/h-BN composite and pure γ-Al_2_O_3_, obtained from the same sol. *—γ-Al_2_O_3_ cF56 (card № 10-0425 ICDD PDF-2 2003); **—γ-Al_2_O_3_ cF8 (card № 75-0278 ICDD PDF-2 2003); ***—γ-Al_2_O_3_ cF16 [24,25]; 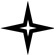
—h-BN.

Figure 3 shows the XRD patterns of a prepared γ-Al_2_O_3_@h-BN composite and pure γ-Al_2_O_3_, obtained from the same sol (inset). A comparison of alumina crystallites formed during the heat treatment of the Al-containing sol and alumina crystallites formed on the h-BN surface shows some differences in their qualitative and quantitative compositions.

The pure γ-Al_2_O_3_ consisted of two syngony species, cF56 and cF8, assigned according to card Nos. 10-0425 and 75-0278 of the ICDD PDF-2 2003 database, respectively. In Figure 3, the syngony species cF56 and cF8 are indicated by the symbols * and **, respectively. Both modifications correspond to the space group Fm3m, whose anions form a face-centered cubic lattice. The medium sizes of the crystallites from the cF56 and cF8 modifications, as calculated using the Rietveld method, were 3 and 7 nm, respectively. The proportions of alumina in the cF56 and cF syngony species were 60 and 40 wt.%, respectively, and thus, the weight ratio of alumina in the cF56 and cF8 syngony species was 1.5.

**Figure 4:**
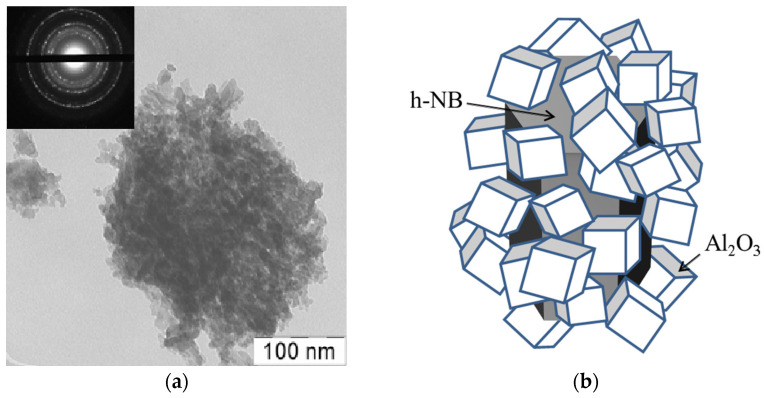
TEM image for pure nano-alumina and electron diffraction (inset) (**a**); scheme of shell formation from γ-Al_2_O_3_ on h-BN crystalline particles (**b**).

According to the TEM data, alumina consisted of cubic nanocrystals less than 10 nm in size (Figure 4a), which is in good agreement with the XRD data. At the same time, the high dispersity of crystallites with cubic syngony was shown in the electron diffraction pattern (Figure 4a, inset).

The XRD data for the composite obtained through the deposition of γ-Al_2_O_3_ on the h-BN powder from an Al-containing sol differed from the data for pure alumina, as shown in Figure 3. As in the case of pure γ-Al_2_O_3_, most of the alumina (70 wt.%) was crystallized in the form of the cF56 syngony with an average crystallite size of less than 3 nm, as calculated using the Rietveld method. The rest of the alumina was crystallized in the form of the cF16 syngony with an average crystallite size of 8 nm. In Figure 3, the syngony species cF16 is indicated by the symbol ***, and it was assigned according to the information presented in [34,35]. Thus, it can be noted that when the crystallization of alumina occurred in the presence of h-BN, the formation of the cF56 syngony occurred more actively, apparently due to the fact that the process occurred on the h-BN surface. At the same time, γ-Al_2_O_3_ appeared in the cF16 system, which was a spinel like the cF8 system in pure γ-Al_2_O_3_.

A comparison of the crystal lattice parameters of two types of syngony showed that the cF56 lattice was deformed during the formation of γ-Al_2_O_3_ on the surface of h-BN crystals, and thus led to the formation of a spinel with the cF16 syngony. The crystal lattice parameter of the cF16 syngony was equal to 3.951 Å, which differs significantly from the corresponding parameter for the spinel cF8, which was equal to 4.096 Å.

In previous works [34,35], a similar phenomenon was observed in the case of the formation of the MgAl_6_O_10_ spinel. During sol–gel synthesis, the covalent interaction of the Al–organic complex with a cation, different from Al^3+^ in size and charge, led to the formation of a spinel with the syngony cF16.

Mild conditions for the synthesis of the γ-Al_2_O_3_@h-BN composite cannot promote the incorporation of B^3+^ ions into the spinel crystal lattice; therefore, it can be assumed that its formation occurs through a surface complex centered on the B^3+^ ion. Apparently, the thermal destruction of the oligomeric gel (Figure 1a), and the subsequent crystallization of alumina on the h-BN surface (Figure 1b), proceed through the formation of a surface complex with the participation of the B^3+^ ion of the surface monolayers of BN crystallites. Figure 4b schematically shows the structure of the nanostructured composite obtained as a result of the deposition of γ-Al_2_O_3_ nanocrystals to h-BN particles.

Thus, we have shown that the direction of alumina crystallization from an oligomeric Al-containing gel on the surface of h-BN particles was determined by B^3+^ ions, which acted as centers of spinel crystallization. In this case, the presence of h-BN promoted a more intense formation of γ-Al_2_O_3_ and an increase in its yield compared to crystallization from a single Al-containing sol. As a result, it was possible to obtain a more homogeneous γ-Al_2_O_3_ phase with a reduced content of spinel.

## 4. Conclusions

Thus, we have proposed a simple, economic, and quick method of obtaining an h-BN@γ-Al_2_O_3_ powder composite with high chemical and phase composition homogeneity, which is intended for sintering special types of ceramics. It was shown that during alumina crystallization on the h-BN surface, the fraction of spinel in alumina decreased from 40 wt.% in pure γ-Al_2_O_3_ to 30 wt.% as a result of the involvement of B^3+^ ions in the nitride surface monolayers to the transition complex. A mechanism has been proposed for the formation of a nanostructured composite γ-Al_2_O_3_@h-BN during the interaction of Al-containing sol and h-BN suspension in organic-aqua media, according to which, alumina crystallization occurred on the B^3+^ centers. The use of the proposed method for obtaining the h-BN@γ-Al_2_O_3_ composite is promising for the creation of new technologies for the production of raw materials for a wide range of purposes.

## Figures and Tables

**Table 1:** Purity and manufacturers of the reagents used.

Raw Materials	Purity	Manufacturer, Country
h-BN powder	>99.00%	Plasmotherm Ltd., Moscow, Russia
Aluminum nitrate nonahydrate Al(NO_3_)_3_·9H_2_O	>97.00%	GOST 3757-75, ChimMed, Moscow, Russia
Monoethanolamine	99.40%	TU 2632-094-44493179-04, EKOS-1, Moscow, Russia
Isopropanol	99.99%	CAS 67-63-0, Sigma-Aldrich, Darmstadt, Germany
Deionized water	specific conductivity < 1 mcm/cm	Raifil water purification system, GOST 6709-72

## Data Availability

The data described in this article are openly available in previous works.
